# Genetic Strategies for Improving Pig Robustness: Reducing Antibiotic Use Through Enhanced Resilience and Disease Resistance

**DOI:** 10.3390/ani15182753

**Published:** 2025-09-20

**Authors:** László Gombos, László Búza, Ferenc Szabó, László Varga

**Affiliations:** 1 Wittmann Antal Multidisciplinary Doctoral School of Plant, Animal, and Food Sciences, Széchenyi István University, 2 Vár Square, 9200 Mosonmagyaróvár, Hungary; swineconsultant@gmail.com; 2 Institute of Food Chain Science, University of Veterinary Medicine, 2 István Street, 1078 Budapest, Hungary; laszlo.buza@yahoo.com; 3 Department of Animal Science, Széchenyi István University, 2 Vár Square, 9200 Mosonmagyaróvár, Hungary; szabo.ferenc@sze.hu; 4 Department of Food Science, Széchenyi István University, 15-17 Lucsony Street, 9200 Mosonmagyaróvár, Hungary

**Keywords:** robustness, pig breeding, disease resistance, swine health, PRRS, genetic selection, antibiotic reduction, animal welfare, immune traits, sustainability

## Abstract

This review investigates genetic strategies aimed at improving robustness in pigs to enhance disease resistance and reduce reliance on antibiotics. Robustness refers to a pig’s ability to stay healthy and productive under stressful or challenging conditions. The review outlines current breeding practices focused on key traits such as maternal ability, growth, immune function, and survival, and highlights that these robustness-related traits show measurable heritability, making them suitable for genetic improvement. Special attention is given to resistance against porcine reproductive and respiratory syndrome (PRRS), a major disease in swine. We also evaluate breed-specific differences, environmental influences, and immune response profiles, emphasizing their impact on breeding outcomes. The development of robust pig lines emerges as a sustainable strategy to reduce antibiotic dependence and enhance herd health. A distinctive contribution of this work is the integration of genetic robustness and resilience strategies with antibiotic stewardship objectives. We link genomic selection, advanced phenotyping, and targeted management interventions within a One Health framework to outline actionable, system-level pathways for reducing antimicrobial inputs. To our knowledge, this combined genetic and public health perspective has not been comprehensively addressed previously.

## Simple Summary

Antibiotic resistance of bacteria is a growing global concern, and pig farming contributes significantly to this issue. This study examines how breeding strategies can support the development of more robust pigs—animals that are healthier, stronger, and better able to cope with stress and disease. The aim is to reduce the need for antibiotics without compromising productivity. The review focuses on genetic approaches that improve key traits such as survival, growth, maternal ability, and immune function. It also examines how selecting pigs with natural resistance to common diseases can lead to healthier herds. Promising techniques include traditional breeding and gene editing. These tools can help produce pigs that are less prone to illness, offering benefits for both farmers and animals through lower costs, improved animal welfare, and reduced environmental impact. The research highlights that breeding for robustness is not only feasible but also essential for the future of sustainable and responsible pig farming.

## 1. Introduction: Importance of Robustness in Pig Production

Robustness is increasingly recognized as a key trait in modern livestock breeding, particularly within swine genetics. Leading pig breeding companies are placing growing emphasis on robustness as a central element of their breeding objectives [1,2,3]. This trend reflects the pressing need to produce strong, resilient pigs capable of performing well under a wide range of environmental and management challenges.

**Figure 1:**
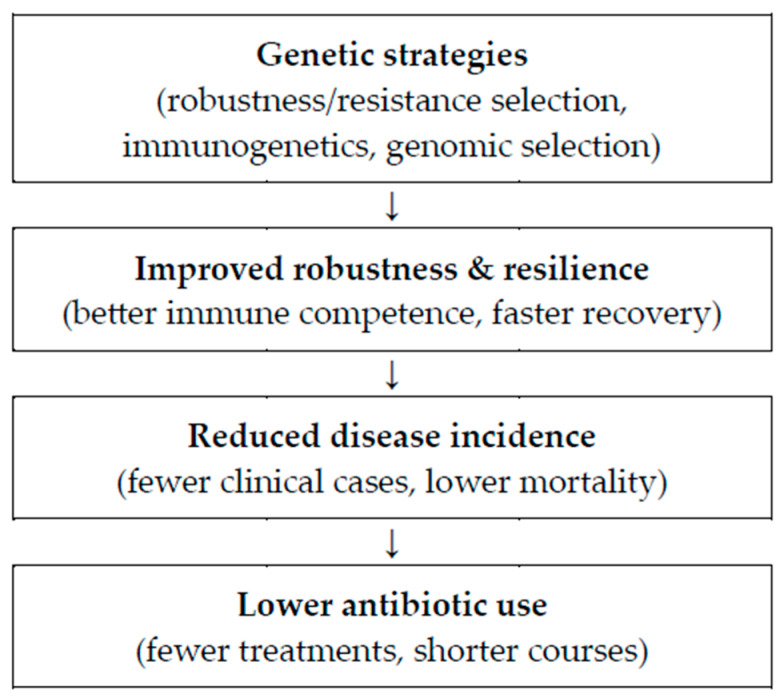
Causal pathway from genetic strategies to reduced antibiotic use in pig production.

High antibiotic use in intensive pig farming is largely a response to the need to control infectious diseases in high-density, high-stress systems where pathogen exposure and limited biosecurity can compromise animal health. However, prolonged and subtherapeutic antibiotic use accelerates the development of antimicrobial resistance by applying selective pressure on microbial populations. This resistance threatens long-term treatment effectiveness, increases morbidity and mortality, and raises serious public health concerns through the risk of zoonotic transmission and environmental contamination. Reducing reliance on antibiotics is therefore critical, and this can be addressed through improved biosecurity, vaccination, management practices, and, notably, genetic selection (Figure 1). Breeding for disease resistance and robustness has emerged as a sustainable strategy to enhance animal health and reduce susceptibility to infections. Research shows that selecting pigs for resilience under infection pressure and improved maternal traits, such as colostrum quality and immunocompetence, results in more robust herds [4,5,6].

Although robustness is a multi-dimensional concept and lacks a universally accepted definition or standardized measurement protocols, it is widely acknowledged as a breeding priority by producers. Increasingly, robustness is being treated as a selection trait alongside traditional production traits. For this integration to be effective, robustness must be clearly defined in performance-related terms and selected in harmony with productivity traits.

Swine genetics companies are now tasked with defining how robustness traits can be meaningfully incorporated into breeding goals and how existing data can be leveraged to quantify the impact of health on productivity. As Knap [4] points out, breeding programs should aim to strike a balance between genetic improvement in productivity and robustness. This approach is especially relevant when addressing sow robustness across markets. Current selection efforts target several priority areas, including sow longevity (e.g., survival through parities 2 and 5, absence of shoulder lesions, and maintenance of body condition), structural health traits (such as osteochondrosis and prolapse incidence), and piglet survival traits, which encompass both mortality causes and physical conformation issues [7].

While this review addresses robustness and resilience in both sexes, particular attention is given to sow traits where maternal effects decisively influence piglet survival and early-life immunity. Assessment of piglet resilience should incorporate early-life identification strategies to enable timely interventions. Combining morphometric measures, such as abdominal circumference and crown–rump length, with birth weight improves prediction of low-viability piglets and informs prompt actions to increase colostrum intake and provide thermal support during the critical neonatal period [8].

This review was based on a structured search of the Scopus database. The Boolean strategy combined the terms (“pig” OR “swine”) AND (“robustness” OR “resilience” OR “disease resistance”) AND (“antibiotic use”). Results were limited to peer-reviewed journal articles, conference papers, books, and book chapters published in English between 1990 and 2025. Eligible studies met the following criteria: peer-reviewed publications in English addressing robustness, resilience, disease resistance, or antibiotic use in swine, and presenting quantitative data, genetic evaluations, or review content relevant to swine breeding. Excluded were non-English publications, non-peer-reviewed material (e.g., conference abstracts, editorials, letters, theses), and studies unrelated to swine genetics or robustness traits.

**Figure 2:**
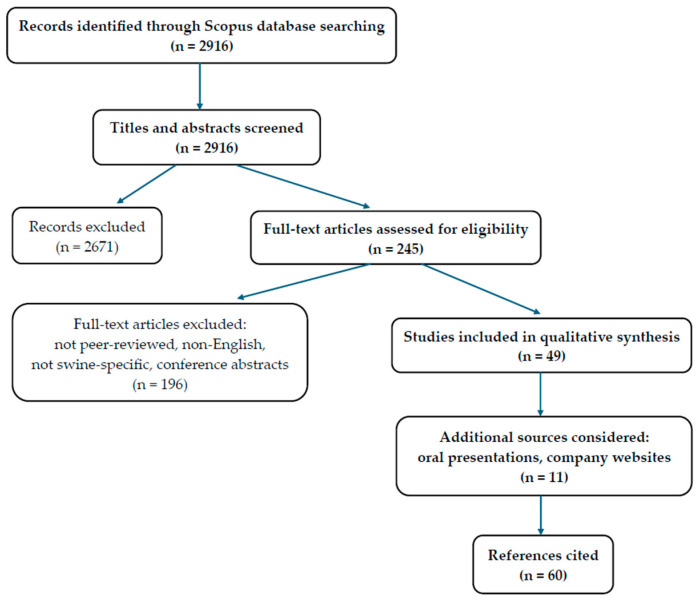
Flow diagram of the study selection process.

The search initially identified 2916 records. After title and abstract screening, 2671 records were excluded. A total of 245 full-text articles were assessed for eligibility, of which 196 were excluded for not meeting inclusion criteria (e.g., not peer-reviewed, non-English, not swine-specific, or conference abstracts). Ultimately, 49 studies were included in the qualitative synthesis. In addition, 11 relevant and recent supplementary sources (e.g., oral presentations at scientific meetings and symposia, as well as information retrieved from major swine breeding companies’ websites) were considered. The selection process is summarized in Figure 2 [9].

## 2. Definition of Robustness in the Context of Pig Breeding

Robustness is the ability of an animal to maintain performance across diverse environmental and management conditions, whereas resilience denotes the speed and extent of recovery after exposure to a stressor. A complementary, operational definition frames robustness as the genetically improved capacity of pigs to grow efficiently, convert feed into weight gain, survive to market weight, and successfully wean numerous high-quality piglets despite routine disease pressure and other stressors. Accordingly, robustness-related traits are commonly grouped into three major categories [3]:

- Sow soundness: Mortality and lameness are primary causes of production losses. Lameness scores contribute to estimated breeding values used in robustness indices.
- Pre-weaned piglets: Traits like birth and weaning weights are critical. Higher birth weight improves survival and future performance.
- Wean-to-finish pigs: Mortality during late finishing is costly. Robust pigs reduce such losses and improve overall efficiency.

In contemporary practice, a competitive maternal line should exhibit five features: (1) high fertility; (2) prolificacy without compromising weaning performance; (3) ease of management; (4) excellent feed efficiency; and (5) superior carcass quality. While often listed alongside these traits, robustness is increasingly central to genetic improvement strategies. Closely related traits include maternal ability, sow longevity, litter size, meat quality, and finisher efficiency. These categories can be further decomposed into measurable indicators such as congenital defects, number of functional teats, leg conformation, shoulder condition, piglet vitality, osteochondrosis, and prolapse occurrence.

**Figure 3:**
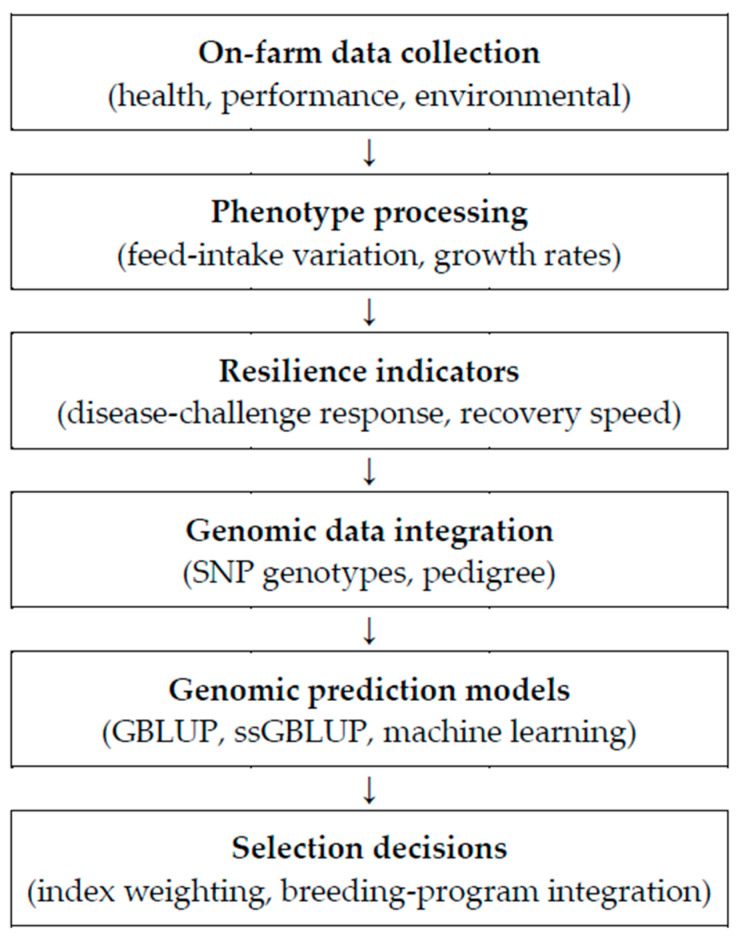
Resilience-aware genomic selection workflow: from on-farm data to breeding decisions.

Advances in livestock breeding increasingly integrate genomic and phenotypic information to enhance resilience and productivity. This framework begins with comprehensive on-farm data collection (health, performance, and environmental variables). Processed phenotypes (e.g., feed-intake variation, growth rate) are complemented by resilience indicators (e.g., response to disease challenge, recovery speed). Genomic information from SNP genotyping and pedigree data is then incorporated into prediction models—genomic BLUP (GBLUP), single-step GBLUP (ssGBLUP), and machine-learning approaches—to generate accurate estimates of genetic merit and to inform index weighting and breeding program integration [10,11]. Collectively, this multilayered approach increases the precision and efficiency of selection, supporting the development of more resilient and productive pig populations (Figure 3).

The breeding companies below are highlighted as representative examples because they publicly document breeding objectives, hold substantial global market share, and explicitly incorporate robustness-related traits into their selection indices.

Topigs Norsvin’s TN70 sow breeding objective targets a highly prolific, self-sufficient sow with strong maternal ability. The breeding program encompasses 51 traits grouped into categories that include sow longevity, maternal ability, litter size, finisher efficiency, carcass and meat quality, and robustness. Notably, robustness receives > 25% of the total selection emphasis and comprises > 50% of the traits by count. Health-related priorities include reducing mortality across all life stages, improving piglet survival, eliminating congenital defects, and decreasing lameness and susceptibility to disease, particularly porcine reproductive and respiratory syndrome (PRRS) [1].

Historically recognized for very large litters, DanBred has addressed the associated management challenges by introducing litter weight gain as a selection trait, serving as a proxy for natural nursing capacity. Emphasizing this trait supports greater milk yield, enables sows to rear a larger proportion of their own piglets, improves litter uniformity, and reduces labor demands in the farrowing unit. In 2025, DanBred reported allocating approximately 66% of its genetic development emphasis to robustness [2].

## 3. Measuring and Scoring Robustness and Resilience

Phenotypic assessment of resilience provides early, noninvasive markers for selection. Deviations in feed intake are sensitive indicators of stress responsiveness and robustness, enabling practical capture of resilience in commercial herds [12]. Moreover, selective breeding for resilience in finishing pigs has been shown to reduce tail biting, lameness, and mortality, demonstrating the welfare and productivity gains from incorporating resilience traits into breeding goals [13].

At the molecular level, blood transcriptome profiling of young, clinically healthy pigs has identified genetic markers associated with disease resilience. These transcriptomic signatures inform immune competence and can be integrated into genomic selection schemes to improve health-related robustness [14].

Scoring is a fundamental approach to measuring robustness traits in pigs. The primary objective is to identify and phenotype resilience-related traits using routinely collected on-farm data, particularly during the fattening period, and to estimate their genetic parameters for use in breeding.

A study conducted from 2019 to 2021 evaluated 7256 pigs from two Piétrain paternal lines at a boar testing station [15]. Throughout the fattening phase (ages 75 to 150 days), individual performance indicators (including growth rate, backfat thickness, loin depth, feed intake, and feed conversion ratio) were recorded, along with observations of suboptimal growth, visible defects, clinical disease symptoms, and records of antibiotic and anti-inflammatory treatments. These indicators were synthesized into three robustness scores:

- R1 identified whether the animal survived the fattening period and reached a minimum weight threshold of 70 kg.
- R2 evaluated the presence or absence of negative health observations, such as poor body condition, abscesses, respiratory issues, or diarrhea. Animals without such issues were classified as “selectable.”
- R3 further refined R2 by distinguishing animals that required medical treatment during fattening from those that remained healthy without any pharmaceutical intervention.

The results demonstrated that pigs scoring higher on robustness indices also exhibited favorable performance traits. Specifically, robustness scores were positively correlated with initial body weight and average daily gain, and negatively correlated with daily feed intake, suggesting that more robust pigs required fewer resources while maintaining performance.

Furthermore, robustness traits R2 and R3 were particularly emphasized for their relevance in assessing pigs’ capacity to sustain performance under environmental and health-related stressors. R2 proved more heritable and required fewer data inputs, making it a practical choice for breeding applications, especially in paternal lines. Although low heritability suggests that genetic progress may be gradual, the inclusion of robustness traits in selection indices adds long-term value beyond conventional performance metrics. R3, while slightly more complex due to its reliance on medication records, could offer deeper insights with improved data collection on breeder interventions. The study highlights the importance of capturing routine health and treatment data as a foundation for developing robust, scalable selection tools. Moreover, both R2 and R3 provide distinct and complementary information, supporting more holistic selection strategies that prioritize resilience alongside productivity [15].

From a genetic perspective, lowering pre-weaning mortality while maintaining or slightly improving birth weight and its uniformity is essential. Balanced, healthy litters lay the foundation for overall herd robustness. Genotypes selected solely for high production can overburden the animal’s resource capacity, compromising immune function under stressful conditions [16].

## 4. Limits to Robustness: Environmental, Welfare, and Economic Constraints

Enhancing genetic potential in livestock must be accompanied by corresponding advances in animal nutrition and management to ensure its full expression. However, these improvements are often underemphasized or overlooked. Certain genetic lines produce progeny with increased muscle mass, which can inadvertently compromise robustness, especially under health challenges or unstable environmental conditions. These animals may struggle to maintain a balance among biological functions, including immune responses. As Knap [4] points out, it is self-evident that improvements in genetic potential must be matched by environmental adjustments that permit effective expression.

Targeted nutritional strategies that enhance sow health and the maternal transfer of immunity can markedly improve piglet survival and robustness. Supplementing sows with multi-species probiotics during late gestation has been shown to increase colostral IgA concentrations, strengthen passive immunity, and reduce preweaning mortality in herds challenged by porcine epidemic diarrhea virus [17]. Maternal antioxidant transfer provides a complementary mechanism: β-carotene and superoxide dismutase act synergistically to cross the placenta and enrich colostrum, improving oxidative balance, piglet growth, and sow body-condition outcomes [18].

Genetic studies further reveal strong correlations between robustness and feed-intake stability under disease pressure, indicating that robust pigs maintain consistent performance even in adverse conditions. These genetic correlations suggest that lower variability in feed intake is associated with reduced mortality and treatment needs and improved growth performance during the finishing phase. Thus, selecting for feed-intake stability under stress could serve as a practical proxy for overall robustness [19]. Incorporating such proxies into selection objectives supports more sustainable breeding programs [5].

Robustness is increasingly associated not only with performance but also with animal welfare. Producers need animals that are strong and healthy—not those that require excessive care or fail to thrive. From a welfare and labor perspective, weak animals represent a discouraging burden for farm staff [20].

Beyond animal welfare, the failure of an animal to perform represents a significant economic loss. The costs of production, housing, and maintenance are high, and every animal must contribute productively to justify these investments. Mortality and morbidity demand additional veterinary treatments and labor, further increasing production costs. Consequently, robustness is not only a welfare concern but also a major economic driver [3]. Moreover, robustness is tightly linked to sustainability, as more robust animals require fewer resources, such as feed and medical interventions, for efficient production.

## 5. Breeding for Health, Stress Resilience, and Performance

Traits currently included in breeding goals that are associated with robustness often relate to health, such as disease resistance, mortality reduction during specific life stages, and reproductive longevity [4,5,21]. Producers do not want to witness premature loss in their herds. Rather, they aim to be responsible stewards of animal welfare and productivity.

At the same time, pig breeders are increasingly faced with diverse and challenging disease pressures. Finding economically viable solutions to maintain animal performance under these conditions is becoming increasingly important [4]. While some genetic systems demonstrate excellent productivity, robustness remains a critical trait, especially for commercial producers worldwide. For instance, if a system produces over 32 piglets per sow per year, the early removal of one sow may equate to the loss of more than 32 marketable pigs [3].

High-performing genetic lines may be more sensitive to environmental stress, making robustness—defined as the ability to maintain high performance while withstanding stressors—a key target in breeding programs [4]. Stress not only reduces productivity but also raises animal welfare concerns. Aggressive behavior, for example, can injure pen mates and suppress overall performance.

To breed for improved behavioral resilience, it is essential to understand the genetic basis of stress-related traits. Estimates of heritability and genetic correlations for behavior traits are necessary before implementing effective selection programs. In a study, behavioral traits were evaluated in a pedigreed Landrace–Duroc–Yorkshire composite population using multi-trait models. All heritability estimates were significantly greater than zero, confirming a genetic basis for behavioral responses. Stress involves complex physiological, neuroendocrine, and behavioral changes, so reducing the frequency and severity of stressors can improve animal health and performance [22].

Robustness goals may vary across regions depending on local production challenges. In some countries, high disease pressure, labor shortages, and reliance on low-cost diets in large-scale systems are the primary concerns. In others, challenges include feed costs, vaccine expenses, heat stress, and treatment efficacy. Long-term goals worldwide increasingly focus on reducing antibiotic use, improving welfare, and promoting environmentally sustainable production systems [23].

A trial comparing two pig genotypes (European and U.S.) across four infection-level environments demonstrated significant genotype-by-environment interactions. Under high-health conditions, certain genotypes performed better, whereas under less supportive conditions, the more robust genotype exhibited superior growth. This highlights the importance of matching genetic selection with environmental context [24].

## 6. From Sows to Piglets: Maternal Effects on Early-Life Survival and Immunity

Improving the health status of a herd begins with the sow. Research shows that piglets born to sows with high reproduction breeding values are more likely to stand promptly, locate a teat quickly, and ingest colostrum faster than piglets from low-breeding-value sows. These early behaviors are critical to survival, highlighting the significant maternal effect on piglet viability. To evaluate sow traits under standardized conditions, trials should be conducted in herds with optimal health and management, where environmental stressors are minimized. Alternatively, data from commercial farms lacking standardized measurements present limitations. As the saying goes: “You can’t improve what you don’t measure.” While genetic evaluations from nucleus farms may exist, robustness-focused selection requires data from commercial environments to account for environmental variability. Therefore, deriving breeding values for robustness traits should involve both nucleus and commercial populations [23].

In recent years, sow milk production has received increasing attention, particularly with regard to both yield and quality. Two traits play a crucial role in determining piglet health: teat morphology and the concentration of immunoglobulins in colostrum. Teat structure affects the efficiency of nursing, while immunoglobulin G (IgG) levels in colostrum are vital for passive immunity and the early development of the piglet’s immune system. One study assessed the heritability of udder traits, specifically morphology and colostral IgG concentration at farrowing, and examined their genetic and phenotypic correlations with reproductive and performance traits. The measured variables included teat length, teat diameter (DIA), inter-teat distance within a row (SAMER), and the teat’s distance from the abdominal midline (AML). These traits demonstrated moderate to high heritability and showed strong correlations with both reproductive and production outcomes. Notably, teat length and DIA were among the most heritable traits [25].

## 7. Integrating Robustness Traits into Genetic Selection Schemes

The heritability of total teat number aligns with previously reported ranges (h^2^ = 0.10–0.42) [26,27]. Udder morphology traits showed high heritability (e.g., h^2^ = 0.46 for teat length, h^2^ = 0.56 for DIA) and moderate values for SAMER (0.37), AML (0.22), udder development score (0.25), proportion of nonfunctional teats (0.30), perpendicular teat orientation (0.10), and colostrum IgG concentration (0.35). These findings underscore the possibility to incorporate these traits into selection indices, appropriately weighted against other economic traits. Notably, the number of piglets born alive but dead within 10 days was positively correlated with DIA, SAMER, and udder development score, and negatively correlated with total teat number [25].

Herd health status is influenced not only by pathogen exposure but also by non-infectious environmental conditions and genetically determined intrinsic factors. Robustness-related breeding goal traits may include conformation traits, osteochondrosis, congenital defects, piglet vitality, mortality at 21 or 28 days, weight at 21 or 28 days, wean-to-rear mortality, and rear-to-finish mortality [23]. A promising approach is to quantify the sensitivity of an animal’s production performance to health challenges across environments. These values can be aggregated for groups and used to support genetic selection. However, implementing such a system presents several challenges [4]:

- Establishing comprehensive data collection across diverse environments;
- Developing robust data processing tools;
- Defining appropriate breeding objectives and selection criteria.

Improved maternal ability directly enhances piglet robustness. Selecting for various traits measured at the individual pig level supports genetic progress from birth to market. Although breeding for robustness is feasible, it requires significant investment in phenotyping infrastructure and genetic technologies. Breeding for increased robustness must be implemented in balance with breeding for increased production. As with all low-heritability traits, DNA information can aid selection; however, this depends on well-designed association studies and application of existing genomic resources in breeding programs [4].

Accurate traceability is equally essential. Each pig must be linked to its sire and dam, enabling meaningful pedigree data collection. Such data supports genetic improvement efforts, particularly at elite nucleus sites, where targeted selection is most impactful [3].

## 8. Integrating Immunogenetics into Pig Breeding: Maternal Effects and Early Immunity

The immune system is a complex and essential biological network that plays a critical role in animal health and is a key consideration in modern pig breeding programs. These programs aim to enhance disease resistance and overall resilience. Genetic studies suggest that immune traits are influenced by both polygenic factors and environmental interactions. While selection for high productivity may inadvertently compromise immune function, improving immunocompetence remains a primary breeding objective [6,28].

Numerous factors affecting piglet immunity have been documented in the literature [29]. In the early postnatal period, maternal effects exert a strong influence on both innate and adaptive immunity, with significant consequences for piglet survival [30]. Since piglets receive maternal antibodies exclusively through colostrum (Table 1), passive humoral and cellular immunity is vital for early protection [31]. Maternal genetic effects also directly impact survival outcomes [32]. As mortality is highest in the early stages of life, even short-duration maternal and litter effects can have long-lasting consequences [33]. Maternal influences modulate key parameters including birth weight, farrowing outcomes, and pre-weaning survival [32].

Innate immunity, the body’s first line of defense against invading pathogens, functions through pattern recognition receptors that detect pathogen-associated molecular patterns. This recognition mechanism enables the immune system to differentiate between self and non-self components [35]. Pathogen-associated molecular patterns are highly conserved molecular structures found across groups of pathogenic microorganisms and include lipids, proteins, and nucleic acids—such as lipopolysaccharides, lipoteichoic acid, and bacterial DNA [36,37,38,39]. These molecules are essential for the survival of pathogens and display molecular or structural characteristics that are absent in host cells. As a result, innate immune cells use pattern recognition receptors to reliably recognize pathogen-associated molecular patterns, trigger immune activation, and help preserve immunological homeostasis [40]. Enhancing the efficiency and specificity of this recognition mechanism through targeted breeding strategies may contribute to improved herd health, increased resilience, and enhanced resistance to infectious diseases.

## 9. Breed-Specific Genetic Variation in Immune Traits of Swine

A large-scale study analyzed the genetic basis of immune traits in Landrace and Large White piglets and their dams by evaluating 22 blood parameters in 1144 piglets aged 6–7 weeks. The study assessed immune cell populations, red blood cell indices, and cytokine profiles. Heritability estimates (h^2^), common environmental effects (c^2^), and genetic correlations (r_g_) were moderate to high, indicating that immune traits are genetically influenced and amenable to selection. Notably, the neutrophil-to-lymphocyte ratio differed considerably between Landrace and Large White breeds [6].

Additional breed-specific differences were observed in innate immune traits. For example, Meishan pigs exhibited higher neutrophil and monocyte counts but lower lymphocyte counts than Large White pigs. Although the optimal balance of immune cells for disease resistance is not fully understood, such differences likely influence pathogen responsiveness [41].

Environmental and physiological factors also play a significant role [41,42]. Variables such as age, housing conditions, and physiological state (e.g., pregnancy or lactation) affect hematological parameters. For instance, lactating sows exhibited higher neutrophil counts and lower lymphocyte counts compared to pregnant sows [43]. Maternal factors—including genotype, parity, and physical condition—also impact piglet immune development, particularly through colostrum quality and intake [44].

Piglet survival and robustness during the suckling period depend on both maternal contributions and genotype–environment interactions. Studies of plasma metabolite dynamics indicate that genetic background and environmental conditions jointly influence piglet vitality, suggesting that early-life resilience is shaped by multifactorial interactions [45].

Litter effects contribute substantially to variation in immune parameters. Moreover, correlations among immune traits and related biological networks support the feasibility of selective breeding for improved immune function [6]. Nevertheless, additional research is needed to clarify the relationship between immune traits and overall productivity.

## 10. Breeding Strategies to Enhance Pig Robustness

Multiple approaches can be used to produce more robust pigs. The first, and most established, is traditional selective breeding. This method is time-consuming but generally considered safe. The second approach involves genetic engineering, commonly referred to as the use of genetically modified organisms (GMOs), where the genetic material is altered using biotechnology techniques [23].

Gene editing, a more precise form of genetic engineering, involves altering DNA by introducing specific base pair changes at targeted locations in the genome. However, the definition of a GMO varies significantly across countries, international organizations, and regulatory bodies. Gene editing raises several critical questions: (1) Does the creation of novel genotypes (e.g., PRRS-resistant pigs) interfere with natural biological processes? (2) Could such modifications increase pathogenicity or drive pathogen mutation? (3) Given that disease resistance is often polygenic, is it advisable to introduce multiple edits into various genes? While gene editing presents significant opportunities, it cannot yet be considered a fully safe or comprehensive solution for disease control [23].

A foundational step is the identification of specific genes and biological pathways involved in disease resistance. Once validated, this knowledge can be transferred to breeding companies to develop commercial pig lines resistant to particular pathogens or disease complexes.

Traditional breeding approaches to enhance disease resistance include three main objectives: (1) selecting animals suited to current and future production environments, (2) reducing antibiotic dependence, and (3) serving a global market.

- Breeding for robust performance in diverse production environments can be achieved in two ways, with both approaches requiring extensive phenotypic data and advanced analytical methods [4]:
  ▪ Incorporating fitness-related traits, such as leg conformation, mortality rates, longevity, and disease resistance, into selection indices alongside production traits.
  ▪ Quantifying environmental sensitivity of production traits using estimated breeding values to assess robustness.
- Antimicrobial resistance poses a critical threat to humans, animals, plants, and ecosystems. A major driver of antimicrobial resistance is the misuse and overuse of antimicrobials in livestock production [46]. Non-infectious factors, such as biosecurity, housing, management, and nutrition, strongly influence antibiotic usage. Improved breeding strategies must consider these factors to reduce antimicrobial use [47].
- Economic analysis of breeding traits is essential for ensuring that improvements are cost-effective and scalable to global markets. Cost–benefit evaluations should link biosecurity, management practices, herd health, and antibiotic usage to assess the economic viability of recommended strategies. Improved animal welfare is also key, as poor welfare increases disease susceptibility and may necessitate antibiotic treatment (e.g., due to tail biting).

## 11. Genomic Prediction and Machine Learning Approaches

Genomic prediction has transformed selection for complex traits, including reproduction and resilience. Genome-wide association studies of reproductive traits have identified multiple loci associated with sow resilience, providing valuable tools for genomic prediction [48]. In Rongchang pigs, machine-learning models substantially improved prediction accuracy for reproductive traits compared with traditional statistical approaches [49].

Comparative evaluations indicate that gradient boosting, support vector machines, and deep learning models can outperform conventional genomic best linear unbiased prediction (GBLUP) when predicting complex traits [50]. In commercial populations, prediction accuracy is highly dependent on reference-population design, and ssGBLUP—which integrates pedigree, phenotype, and genotype information—often outperforms alternative models [51]. Collectively, these findings highlight the central role of advanced computational strategies, including machine learning and ssGBLUP, in contemporary pig-breeding programs.

## 12. Genomic Selection

When direct measurement of selection traits is complicated, genomic information (SNPs, genes) provides a powerful alternative. Once a reliable association between DNA profile and a target trait is established, future selection can rely on laboratory genotyping rather than waiting for phenotypic expression. Genomic prediction of robustness and incorporation of large SNP panels into GBLUP are particularly effective for traits with low heritability. A major advantage is that animals need not be exposed to pathogens or withheld from treatment to assess disease resistance. This allows selection for health traits without compromising welfare or biosecurity. Indicator traits with potential for marker-based selection include:

- Pre-weaning survival: stillbirth rate, mortality, birth weight, disease resistance, milk yield, maternal behavior.
- Post-weaning survival: mortality, disease resistance, heat tolerance.
- Sow longevity: number of litters, leg and udder quality, heat tolerance.

These traits often show moderate to strong correlations with key objectives but typically have low heritability (h^2^ ≤ 0.1) and are environmentally sensitive.

In infectious conditions, observed performance traits such as growth reflect both genetic potential for productivity (h^2^ ≈ 0.3) and disease resistance (h^2^ ≈ 0.1). Thus, selecting for production indirectly improves resistance. Over 30 generations, this can increase population-level resistance by 170% from baseline. Remarkably, a single observation of such a trait can provide as much genetic information as a marker panel covering 10% of resistance variance [4].

Significant progress has already been made in identifying DNA markers associated with resistance to specific pathogens, such as *Escherichia coli* F18 [52], *Glaesserella parasuis* [53], and *Salmonella enterica* subsp. *enterica* serovar Choleraesuis [54].

## 13. PRRS Resistance as a Model for Breeding Robust and Resilient Pigs

Eliminating infectious agents such as porcine reproductive and respiratory syndrome virus (PRRSV), *Mycoplasma hyopneumoniae*, and *Actinobacillus pleuropneumoniae*—the principal pathogens contributing to the porcine respiratory disease complex—has the potential to markedly enhance animal health, production efficiency, and the economic sustainability of pig farming systems [47]. Improving resistance to these diseases can reduce intra-herd variability, culling rates, and mortality, while also lowering the reliance on antibiotics and other pharmaceutical interventions [19].

PRRSV is a particularly illustrative case: the disease causes substantial economic losses worldwide, estimated at €14 million annually in Hungary [55] and $664 million in the United States [56]. These figures underscore the necessity of deploying all available tools, both eradication efforts and genetic approaches, to mitigate the disease’s impact.

The value of controlling or eliminating pathogens with minimal or no antibiotic use is further demonstrated by sustainability metrics. During the national PRRS eradication program in Hungary, the sow population decreased by 26.2% while slaughter pig output remained nearly unchanged, resulting in substantial reductions in ammonia emissions, slurry production, nitrogen emissions, and total greenhouse gas emissions (91,768,362 kg CO_2_eq) [57]. Concurrently, farm data highlighted that appropriately designed herd-health programs are pivotal for reducing antibiotic use [58].

Recent work emphasizes the importance of collecting and analyzing data from commercial farms introducing modern, more robust genetics to improve competitiveness, reduce losses from endemic pathogens, strengthen biosecurity, and lower antibiotic use. In one study, improved health status was associated with a 1.95 kg CO_2_eq reduction per kg carcass, an average 60% reduction in antibiotic use with elimination of all “restrict” category antimicrobials, a 60% decrease in offspring losses (up to 90% on some finisher farms), and an average 35% improvement (decrease) in feed conversion ratio [59].

Although Hungary successfully completed a nationwide PRRS eradication program by 2021 [55], long-term, sustainable solutions must include genetic selection for enhanced disease resistance. Direct selection for robustness and marker-assisted selection targeting the WUR SNP on chromosome 4 are promising avenues. Animals carrying the favorable WUR B allele show improved PRRSV resistance, increased average daily weight gain, lower mortality rates, and reduced antibiotic use [60].

Experimental evidence supports robustness as a breeding target. Under PRRSV challenge, litters sired by high-robustness boars performed significantly better: piglet mortality between 0 and 21 days post-inoculation was twice as high in the low-robustness group, and the number of injectable antibiotic treatments was significantly greater (*p* < 0.01) [19].

Importantly, robustness extends beyond PRRSV. More robust animals also exhibit greater resilience to co-infections and environmental stressors, improving production efficiency and reducing medication needs [20]. A study conducted in 2020–2021 identified *Streptococcus suis* serotypes 2 and 1/2 as the primary causes of severe clinical disease and demonstrated that integrating targeted vaccination, strict biosecurity, enhanced colostrum management, and crossbreeding with robust sows effectively controls *Streptococcus suis* infections, reduces antimicrobial dependence, improves piglet growth, and yields significant economic benefits. Results indicate that *Streptococcus suis*-associated mortality decreased by 90%, antibiotic use in suckling and weaned piglets declined by more than 98%, and critically important antimicrobials were eliminated within one year, lowering medication costs by approximately 91% [61].

Collectively, the evidence strongly supports an integrated strategy that combines disease eradication programs with genetic improvement for robustness to build a more sustainable, resilient, and economically viable pig production system.

## 14. Conclusions

Integrating robustness and resilience into pig-breeding programs offers a clear pathway to more sustainable, health-oriented production systems. Future breeding concepts will prioritize animals that maintain performance under environmental and disease-related stress, thereby reducing reliance on antibiotics and other pharmaceutical interventions. Achieving this objective requires coordinated progress across genomic, phenotypic, and nutritional domains.

However, implementation is not without constraints. Ethical, regulatory, and biosafety considerations remain central when applying genetic engineering or genome editing for disease resistance. In robustness-focused selection indices, practical challenges include high phenotyping costs, longer selection cycles, and variable data quality in commercial herds.

Although many studies report positive genetic correlations between resilience indicators and economically important traits, results are not uniformly consistent. Resilience is multidimensional, and trade-offs have been documented. For example, increased resistance to infectious disease has, in some cases, been associated with slower growth. The heritability of immune-response traits remains contentious: some studies find moderate to high heritability for antibody responses and cytokine profiles, whereas others detect minimal additive genetic variance. These discrepancies underscore gaps in understanding the genetic architecture of robustness and highlight the importance of context, including pathogen type, challenge model, and population structure. Large-scale, multi-population studies with standardized phenotyping and challenge protocols are needed.

Despite these challenges, breeding pigs that are both robust and highly productive is feasible and valuable for commercial systems. As demand grows for healthier, antibiotic-reduced, and more sustainable pork, alternative health strategies have become essential. Safeguarding herd health is a shared responsibility that integrates data-driven farm management with forward-looking veterinary care. Advanced genomic tools should be leveraged to monitor population health, strengthen immune competence, and prevent disease outbreaks, enabling animals to realize their genetic potential.

Resilience can be effectively enhanced through a dual strategy: (1) incorporating phenotypic indicators, such as deviations in feed intake and health-related behavior, into breeding goals; and (2) applying advanced genomic prediction methods, particularly machine learning and ssGBLUP, to increase selection accuracy for complex traits. Phenotypic and transcriptomic indicators provide actionable measures of robustness, while modern prediction models improve genetic gain. Integrating these approaches supports more sustainable, welfare-oriented production.

Economic concerns about reduced antibiotic use and alternative health-supporting strategies are often overstated. Rigorous analyses show that practices initially viewed as long-term investments, such as breeding for disease resistance, can yield measurable returns within one or two production cycles through improved health, lower treatment costs, and increased productivity.

Breeding companies play a pivotal role in advancing robustness and resilience alongside productivity. A forward-looking strategy should explicitly incorporate maternal effects, innate immune function, and resilience phenotyping into breeding objectives. Progress in improving resistance to PRRS illustrates the transformative potential of these approaches.

Looking ahead, the most impactful programs will integrate genomic prediction and resilience phenotyping with precision farm management and proactive veterinary medicine. Such frameworks will allow pigs to realize their genetic potential while minimizing health risks and meeting evolving consumer expectations for sustainable, antibiotic-reduced pork.

## Figures and Tables

**Table 1:** Development of the piglet immune system in the early postnatal period [34].

Cell Type/Function	Neonatal Status	Development Timeline	Colostrum Transfer
Phagocytosis	Low	Matures over 12 weeks	–
Neutrophils	High	Decrease until 3 weeks, followed by an increase	Yes
Macrophages	Low	Alveolar by 2 weeks, intravascular by 3–7 days	–
Natural killer cells	Absent	Detected at 2–3 weeks	–
B lymphocytes	Low (3–4%)	Mature by 4 weeks	Yes
T lymphocytes (CD4+/CD8+)	Low (3–4%)	Mature by 4 weeks	Yes
Memory cells (CD4+/CD8+)	Absent	Rapid increase until 6 months, then slows	–
Intestinal lymphoid tissue	Underdeveloped	Develops by 4 weeks	–

## Data Availability

All data underlying this study are publicly available in the cited publications.
